# Primary Breast Mucinous Cystadenocarcinoma and Review of Literature

**DOI:** 10.7759/cureus.23098

**Published:** 2022-03-12

**Authors:** Ambreen Moatasim, Nadira Mamoon

**Affiliations:** 1 Pathology, Shifa International Hospital Islamabad, Islamabad, PAK

**Keywords:** dcis, rare, er, breast, mucinous cystadenocarcinoma

## Abstract

Mucinous cystadenocarcinoma of the breast is a rare primary breast carcinoma having distinct clinical behavior and a favorable prognosis. It has a characteristic morphology that must be differentiated from metastatic ovarian and pancreatic mucinous adenocarcinoma. The etio-pathogenesis, genetic profile, and treatment of this tumor are controversial. Here, we report a case of primary mucinous cystadenocarcinoma of the breast in a 61-year-old female. The case is of interest since it is uncommon and has peculiar clinical and morphological features.

## Introduction

Mucin-producing carcinomas are unusual primary malignancies of the breast and constitute about 1%-4% of total breast cancer. According to the WHO classification, the mucin-producing carcinomas of the breast are divided into four histologic subtypes, including mucinous carcinoma, mucinous cystadenocarcinoma (MCA), columnar cell mucinous carcinoma (CCMC), and signet ring cell carcinoma [1]. MCA usually occurs in elderly women, shows a striking resemblance to MCAs of the pancreas and ovary, is triple-negative, and has a favorable prognosis.

We report an unusual case of primary MCA of the breast in a 61-year-old lady presenting as a cystic mass. The tumor exhibited features like the association with ductal carcinoma in situ (DCIS), lymph node metastasis, and estrogen receptor positivity which are rarely reported in the literature.

## Case presentation

A 61-year-old lady presented to the breast clinic with a complaint of a left-sided breast lump for six months and pain for two weeks. On mammography, the left breast showed a well-defined oval-shaped high-density mass with a cystic component in the retro areolar region associated with architectural distortion. Overall findings were assessed as Breast Imaging Reporting and Data System (BIRADS) category 5 (highly suggestive of malignancy). On the right side, an iso-dense partially circumscribed mass in the subareolar region, lower inner quadrant, was seen which when correlated with ultrasound showed an oval-shaped, hypoechoic mass. Overall findings were assessed as BIRADS category 4a (low suspicion of malignancy). Tissue sampling was suggested.

She underwent a core needle biopsy for both right and left-sided lesions. Histological evaluation showed papillary neoplasm on the left side. Based on morphology and immunohistochemical findings, the differentials included encapsulated papillary carcinoma and papilloma with DCIS. Complete excision of the lesion with clear margins was suggested for further evaluation. The right side showed benign breast parenchyma.

**Figure 1:**
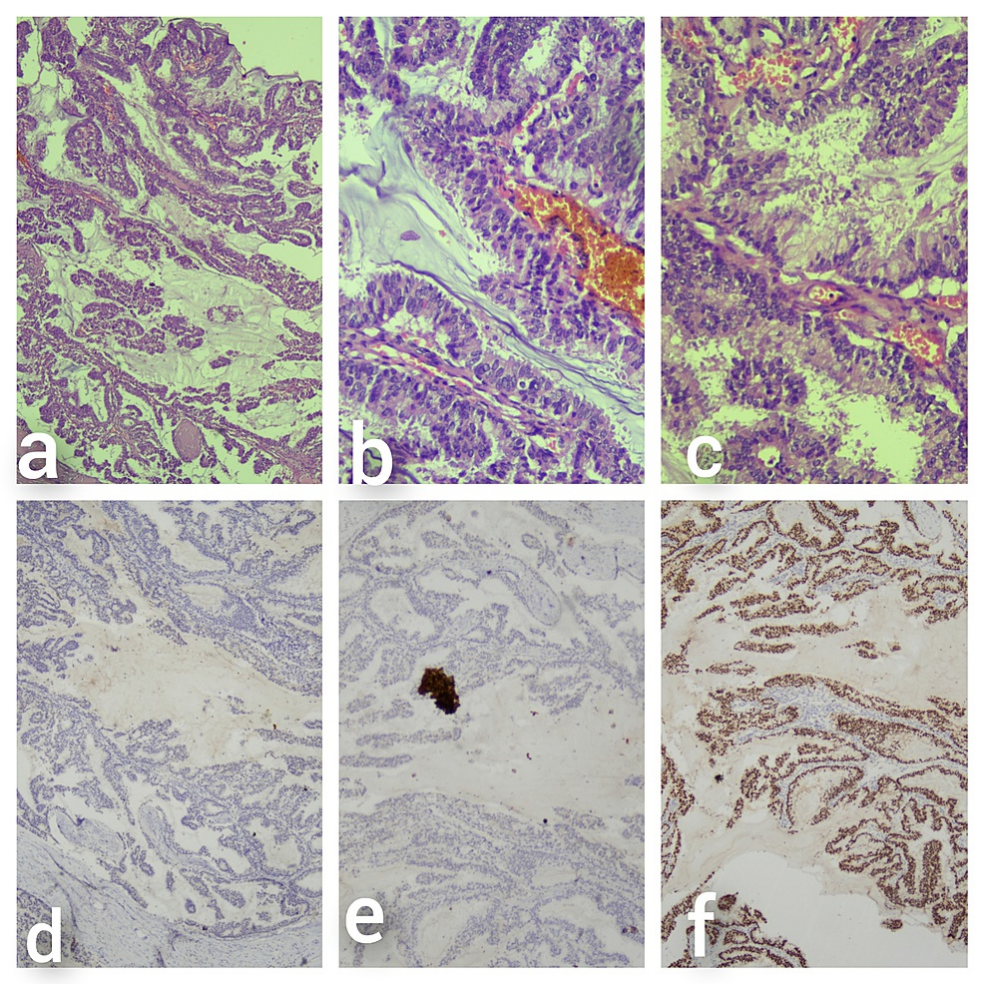
(a) Low power (H&E x4M), (b,c) high power (H&E x10M) showing a papillary neoplasm with extra and intracellular mucin. Immunohistochemistry (d) p63 and (e) CK5/6 are negative while (f) ER is strongly positive.

**Figure 2:**
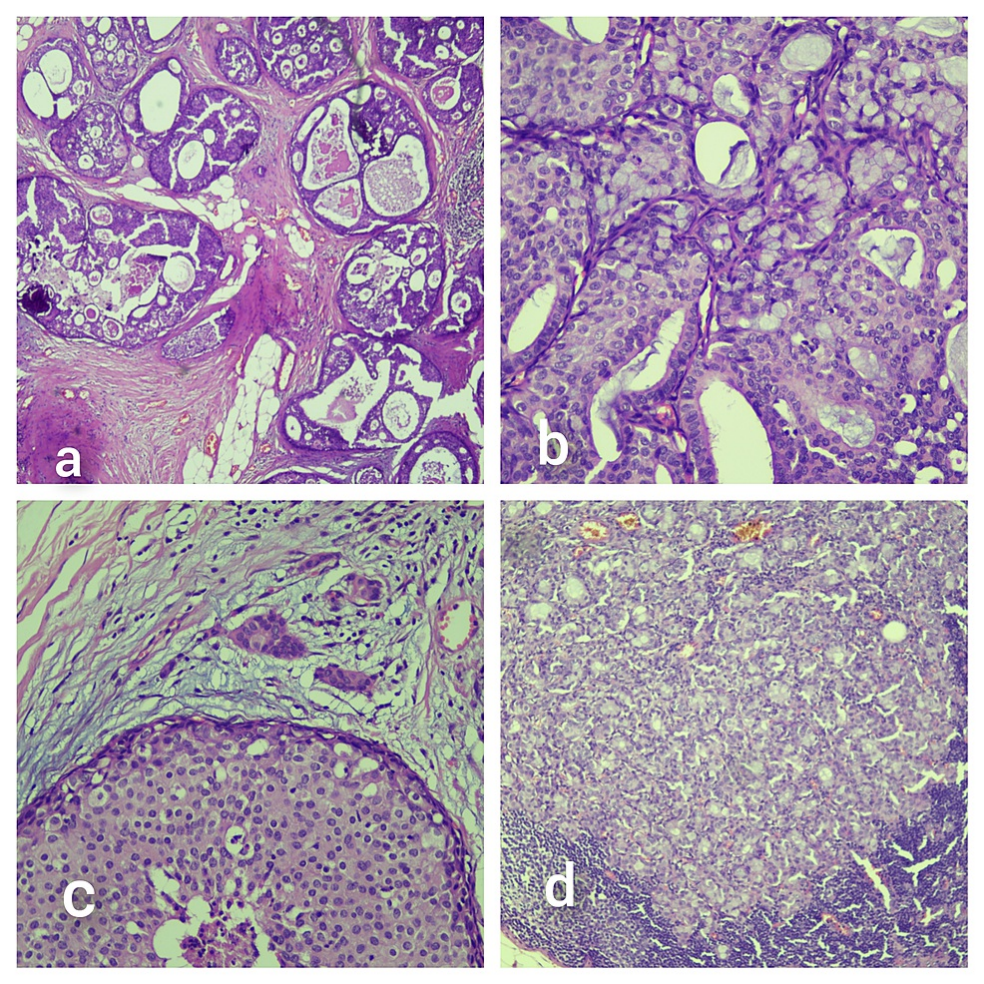
(a) Surrounding DCIS (H&E x4M), (b) intracellular mucin within DCIS (H&E x10M), (c) microinvasion (H&E x10M), and (d) lymph node metastasis (H&E x10M). DCIS - ductal carcinoma in situ

Following MDT consultation, left-sided modified radical mastectomy was performed with level II axillary dissection. The gross examination revealed a 3.5 x 3.0 x 1.0 cm cystic tumor in the central part of the breast. Sections were taken, processed, and stained with H&E. The tumor was a papillary neoplasm lined by stratified columnar epithelium having apical snouts and focal mucinous cytoplasm. p63 and CK5/6 were negative while ER was strong positive (Figures 1a-1f). The tumor was GATA3 positive and negative for CDX2. The adjacent breast showed foci of conventional, intermediate nuclear grade, DCIS. The DCIS was also strongly positive for ER. After thorough processing of the tumor and surrounding area, a focus of microinvasion was also seen (Figures 2a-2d). Four out of 20 lymph nodes recovered showed metastatic carcinoma. Interestingly, mucin was appreciated focally in the tumor as well as extracellularly. Mucin was also seen in foci of metastasis and DCIS. A final diagnosis of MCA was rendered with adjacent DCIS and a focus of microinvasion. The pathologic stage was pT1mi N2a. All margins were uninvolved by invasive carcinoma and DCIS.

Eight months post-surgery, the patient is doing well and has been advised regular follow-up visits.

## Discussion

Mucinous lesions of the breast constitute a large spectrum ranging from benign to invasive mucinous carcinoma [2]. The World Health Organization classification divides mucinous carcinoma into four different subtypes.

Primary MCA is a rare tumor usually occurring in postmenopausal women and was first described by Koenig and Tavassoli in 1998 [3]. At present, less than 30 cases have been reported worldwide and most of them are just case reports [4].

The tumor is mostly multicystic lined by columnar mucinous epithelium with single or pseudostratified basal nuclei that present mild or no atypia. Tumor displays intra and extracellular mucin, tufting, and papillary formations. Desmoplastic stroma may be observed. No obvious mitosis and necrosis are observed. It is difficult to exclude benign or borderline lesions and to make a definite diagnosis of malignancy based on morphology alone. However, the absence of myoepithelium, frequent association with DCIS, and the capability of metastasis suggest malignancy [5].

The primary MCA of the breast is histologically similar to the primary MCA of the pancreas and ovary [3]. Confirmation of the diagnosis requires the exclusion of metastasis from these sites. The tumor cells show a p63-/CK7+/CK20-/CDX2-/villin-pattern, with overexpression of p53 and EGFR. All reported cases of MCA are usually triple-negative with the exception of one case reported by Rakici et al. [6] that was estrogen receptors (ER)-positive. Our case was also ER, progesterone receptors (PR) positive, and human epidermal growth factor receptor-2 (Her2) Neu negative with a very low proliferative index (<10%).

The exact pathogenesis of this tumor is unclear. The coexistence of ordinary DCIS and MCA in some cases indicates that tumor cells of MCA might have been transformed through metaplasia of epithelial cells of DCIS, accompanied by loss of expression of estrogen and progesterone receptors [7-9]. According to Lee and Chaung [10], the neoplasm is the result of an accumulation of mucin within intraductal papillary carcinoma with mucinous metaplasia and extracellular mucin production. This causes cystic dilation of the lumen with loss of myoepithelial cells and invasion of the adjacent stroma.

Lymph node metastasis is infrequent but is reported in the literature. The prognosis despite lymph node metastasis is favorable with no recurrence or distant metastasis after complete resection. Our case showed metastases in four out of 20 lymph nodes recovered. So far, the patient is on regular follow-up with no recurrence or development of metastasis.

The usual treatment is complete resection, mastectomy, or lumpectomy, followed by axillary lymph node dissection. The utility of neoadjuvant therapy or radiation is debatable.

## Conclusions

In conclusion, we report a rare case of MCA of the breast. It must be diagnosed after excluding metastatic tumors from other sites, particularly the pancreas and ovary. Our case showed some distinctive features like ER and PR receptor positivity as well as lymph node metastasis which are rarely reported in the literature. Awareness of this entity is necessary since it has a good overall prognosis with no reports of local recurrences or distant metastasis following adequate treatment.
